# Prognostic significance of the radiologic features of pneumonitis induced by anti‐PD‐1 therapy

**DOI:** 10.1002/cam4.2974

**Published:** 2020-03-09

**Authors:** Satoshi Watanabe, Takeshi Ota, Masachika Hayashi, Hiroyuki Ishikawa, Aya Otsubo, Satoshi Shoji, Koichiro Nozaki, Kosuke Ichikawa, Rie Kondo, Takao Miyabayashi, Satoru Miura, Hiroshi Tanaka, Tetsuya Abe, Masaaki Okajima, Masaki Terada, Takashi Ishida, Akira Iwashima, Kazuhiro Sato, Hirohisa Yoshizawa, Toshiaki Kikuchi

**Affiliations:** ^1^ Department of Respiratory Medicine and Infectious Diseases Niigata University Graduate School of Medical and Dental Sciences Niigata Japan; ^2^ Niigata Prefectural Shibata Hospital Niigata Japan; ^3^ Department of Diagnostic Radiology Niigata University Medical and Dental Hospital Niigata Japan; ^4^ Niigata City General Hospital Niigata Japan; ^5^ Niigata Cancer Center Hospital Niigata Japan; ^6^ Shinrakuen Hospital Niigata Japan; ^7^ Saiseikai Niigata Hospital Niigata Japan; ^8^ Niigata Prefectural Central Hospital Joetsu Japan; ^9^ Nagaoka Chuo General Hospital Nagaoka Japan; ^10^ Nagaoka Red Cross Hospital Nagaoka Japan; ^11^ Niigata Medical Center Niigata Japan

**Keywords:** immune checkpoint inhibitors, immune‐related adverse event, interstitial lung disease, NSCLC, PD‐1

## Abstract

**Background:**

Interstitial lung disease (ILD) induced by anti‐programmed‐cell death‐1 (PD‐1) and anti‐PD‐ligand 1 (PD‐L1) is potentially life‐threatening and is a common reason of the discontinuation of therapy. In contrast, an enhancement in antitumor effects was reported in patients who developed immune‐related adverse events, including ILD. Although recent evidence suggests that radiologic patterns of ILD may reflect the severity of ILD and the antitumor immune responses to anti‐PD‐1/PD‐L1 therapies, the association between radiologic features and clinical outcomes remains unclear.

**Methods:**

Patients with advanced non‐small‐cell lung cancer who were treated with 1st to 3rd line anti‐PD‐1 therapy from January 2016 through October 2017 were identified at multiple institutions belonging to the Niigata Lung Cancer Treatment Group. ILD was diagnosed by the treating physicians, and chest computed tomography scans were independently reviewed to assess the radiologic features of ILD.

**Results:**

A total of 231 patients who received anti‐PD‐1 therapy were enrolled. Thirty‐one patients (14%) developed ILD. Sixteen patients were classified as having ground glass opacities (GGO), 16 were classified as having cryptogenic organizing pneumonia (COP), and one was classified as having pneumonitis not otherwise specified. Patients with GGO had significantly worse overall survival time compared to patients with COP (7.8 months (95% CI: 2.2‐NE) versus not reached (95% CI: 13.2‐NE); *P* = 0.0175). Multivariate analysis of all 231 patients also revealed that PS = 1 and ≥2 and GGO were significant predictors of a worse overall survival.

**Conclusions:**

This study demonstrated that patients who developed GGO exhibited worse outcomes among non‐small‐cell lung cancer patients receiving anti‐PD‐1 therapies.

## Graphical abstract

Interstitial lung disease (ILD) induced by anti‐programmed‐cell death‐1 (PD‐1) is potentially life‐threatening and radiologic patterns of ILD may reflect the severity of ILD and the antitumor immune responses to anti‐PD‐1 therapies. The current study demonstrated that patients who developed ground glass opacities exhibited worse outcomes among non‐small‐cell lung cancer patients treated with anti‐PD‐1 therapies.

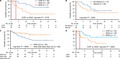

**Abbreviations**
COP: cryptogenic organizing pneumonia
GGO: ground glass opacities
ICI: immune checkpoint inhibitor
ILD: interstitial lung disease
irAEs: immune‐related adverse events
NOS: not otherwise specified
NSCLC: non‐small‐cell lung cancer
OS: overall survival
PD‐1: programmed‐cell death‐1
PD‐L1: PD‐ligand 1
PFS: progression‐free survival

## 1 INTRODUCTION

Immune checkpoint inhibitors (ICIs), including anti‐programmed‐cell death‐1 (PD‐1) and anti‐PD‐ligand 1 (PD‐L1) antibodies, have demonstrated promising and durable benefits in non‐small‐cell lung cancer (NSCLC) patients.^1^, ^2^, ^3^, ^4^, ^5^ Anti‐PD‐1 and anti‐PD‐L1 antibodies have become the new standard of care for NSCLC patients. Because interactions between PD‐L1and PD‐1 maintain immune tolerance to normal tissues peripherally, blockade of PD‐L1 and PD‐1 results in the activation of the immune system and often causes immune‐related adverse events (irAEs).^6^ Previous studies have shown that clinical efficacies of anti‐PD‐1/PD‐L1 therapies were augmented in patients with irAEs, suggesting that anti‐PD‐1/PD‐L1 antibodies are capable of stimulating immune responses against both normal cells and tumor cells.^7^, ^8^

Previous phase III studies have reported that the incidence of interstitial lung disease (ILD) induced by anti‐PD‐1/PD‐L1 therapies was 1%‐6%.^1^, ^2^, ^3^, ^4^, ^5^ ILD is potentially fatal and often results in the discontinuation of therapy.^3^, ^9^ Indeed, several studies have shown that ILD was the most common irAE leading to the discontinuation of anti‐PD‐1 therapies.^1^, ^2^ On the other hand, the augmentation of antitumor effects was reported in patients with ILD, similar to other irAEs.^10^, ^11^, ^12^

Several radiologic patterns of ILD caused by ICIs have been reported.^13^, ^14^, ^15^ Although these radiologic patterns may reflect the severity of ILD and the antitumor immune response enhanced by anti‐PD‐1/PD‐L1 therapies, the correlation between radiologic patterns and clinical outcomes, especially the augmentation of antitumor effects, remains unclear. The aim of this study is to elucidate the correlation between the radiologic features of ILD induced by anti‐PD‐1 antibodies and clinical course of patients with advanced NSCLC.

## 2 PATIENTS AND METHODS

### 2.1 Study design and patients

The medical records of all consecutive patients with advanced NSCLC who were treated with anti‐PD‐1 as 1st to 3rd line therapy at multiple institutions belonging to the Niigata Lung Cancer Treatment Group from January 2016 through October 2017 were retrospectively reviewed. We started this study after approval from the institutional review board of each participating institution.

### 2.2 Study assessment

For all patients, the following data were collected retrospectively: demographics, oncologic therapy including anti‐PD‐1 therapy, and irAEs. For patients with ILD, the clinical features of ILD and treatment for ILD were obtained. ILD was diagnosed by the treating physicians. The chest computed tomography (CT) scans of patients with ILD were obtained and independently reviewed by two pulmonologists (MH and SW) and one radiologist (HI) blinded to patient clinical data. ILD was classified into 5 subtypes: cryptogenic organizing pneumonia (COP), ground glass opacities (GGO), interstitial, hypersensitivity and pneumonitis not otherwise specified (NOS) according to previous reports.^14^, ^16^, ^17^, ^18^ If there was disagreement, a consensus read was performed. ILD was graded according to the Common Terminology Criteria for Adverse Events version 4.0. Progression‐free survival (PFS) was calculated from the start of anti‐PD‐1 therapy until progressive disease or death due to any cause. Overall survival (OS) was measured as the time between the start date of anti‐PD‐1 therapy and death due to any cause.

### 2.3 Statistical analysis

Kaplan‐Meier survival curves were constructed for PFS and OS, and differences between groups were identified using the log‐rank test. Continuous variables are presented as the median (range) and were compared by two‐sided *t* tests. Categorical variables were compared by Fisher's exact test or chi‐square test. To evaluate prognostic factors for OS, we used multivariate Cox proportional hazards models. Multivariable analysis was performed, including age, sex, smoking status, histology, Eastern Cooperative Oncology Group performance status, irAE and radiologic features. To minimize lead‐time bias associated with time‐dependent factors, we performed landmark analysis including only patients who were alive or whose disease was under control at 43 days after anti‐PD‐1 therapy, which is the median time of onset of ILD, for OS (n = 214) and PFS (n = 172). Additionally, we performed landmark analysis at 6 weeks after anti‐PD‐1 therapy to evaluate the difference in OS between patients with and those without irAE using multivariate Cox proportional hazards models. All the reported p‐values were 2‐sided, and *P* < 0.05 was considered significant. Statistical analysis was performed using JMP 9.0.2 statistical software (SAS Institute, Cary, NC).

## 3 RESULTS

### 3.1 Patient characteristics

**FIGURE 1:**
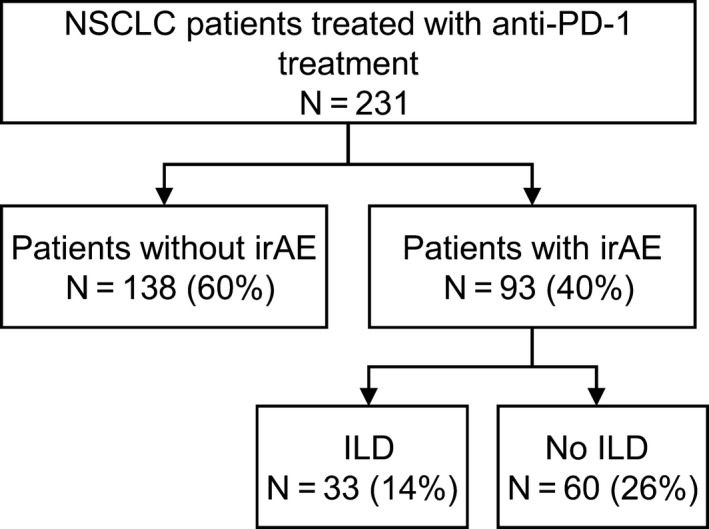
Patient flow diagram

Overall, 231 patients were enrolled in this study. Among these patients, 33 patients (14%) developed ILD (CONSORT diagram [Figure 1]). Baseline characteristics at the initiation of anti‐PD‐1 therapy for patients with and those without ILD are presented in Table 1. The frequency of squamous carcinoma was significantly higher in patients with ILD than in patients without ILD. There were no significant differences in terms of age, sex, smoking status, Eastern Cooperative Oncology Group performance status, line of anti‐PD‐1 therapy and type of anti‐PD‐1 antibody.

**TABLE 1:** Patient Characteristics at PD‐1 therapy

Clinical feature		Without ILD (total, n = 198)	With ILD (total, n = 33)	*P* value
Median age, years (range)		68 (38‐84)	66 (45‐82)	.4941^a^
Sex, n (%)	Female/male	48 (24)/150 (76)	7 (21)/26 (79)	.8271^b^
Smoking status, n (%)	Current or former	157 (79)	29 (88)	.3435^b^
	Never	41 (21)	4 (12)	
PS, n (%)	0	46 (23)	9 (27)	.5653^c^
	1	115 (58)	16 (48)	
	≥2	36 (18)	7 (21)	
	Unknown	1 (1)	1 (3)	
Stage, n (%)	IIIB	21 (11)	1 (3)	.2818^c^
	IV	101 (51)	20 (61)	
	Relapse after local therapy	76 (38)	12 (36)	
Histology, n (%)	Adenocarcinoma	120 (61)	12 (36)	.046^c^
	Squamous carcinoma	63 (32)	17 (52)	
	Others	15 (8)	4 (12)	
Line of anti‐PD‐1 therapy, n (%)	1	31 (16)	6 (18)	.8484^c^
	2	102 (52)	17 (52)	
	3	65 (33)	10 (30)	
PD‐L1 expression, n (%)	<1	14 (7)	1 (3)	.7422^c^
	1%‐49%	12 (6)	1 (3)	
	>50%	46 (23)	8 (24)	
	Unknown	126 (64)	23 (70)	
Anti‐PD‐1 therapy, n (%)	Nivolumab	151 (76)	25 (76)	1^b^
	Pembrolizumab	47 (24)	8 (24)	
Radiologic features, n (%)	COP‐like		16 (48)	
	GGO		16 (48)	
	Not otherwise specified		1 (3)	

Note

Differences between groups were identified using:

Abbreviations: COP, cryptogenic organizing pneumonia; GGO, ground glass opacities; ILD, interstitial lung disease; PD‐L1, PD‐ligand 1; PD‐1, programmed‐cell death‐1; PS, performance status

^a^ student's *t *test

^b^ Fisher's exact test

^c^ Chi‐Square test.

### 3.2 Radiographic patterns of ILD and OS

**FIGURE 2:**
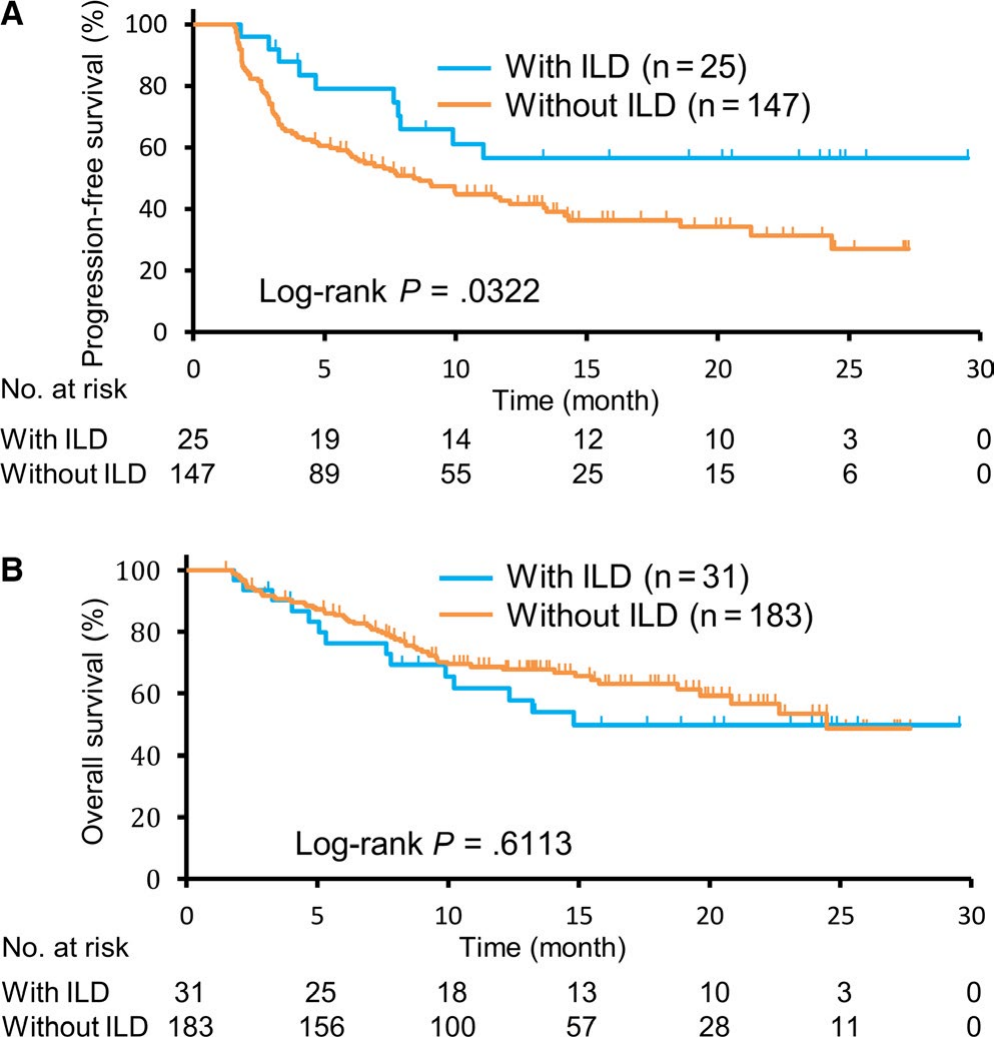
The progression‐free survival curves (A) and overall survival curves (B) of the patients with or without ILD. ILD, interstitial lung disease

The Kaplan‐Meier curves for PFS for patients with and without ILD are shown in Figure 2A. The median PFS among patients with ILD was significantly longer than that among patients without ILD (not reached (95% CI: 7.8‐NE) vs 8.6 months (95% CI: 5.9‐13.4); *P* = 0.0322). However, similar OS times were observed between patients with ILD and those without ILD (14.8 months (95% CI: 7.8‐NE) vs 24.5 months (95% CI: 19.6‐NE); *P* = 0.6113) (Figure 2B).

### 3.3 Baseline characteristics in patients with ILD

Of 33 patients with ILD, 16 were classified as having GGO, 16 were classified as having COP, and one was classified as having pneumonitis NOS (Table 1). Representative chest CT scans of patients with GGO, COP and pneumonitis NOS are shown in Figure S1. Table 2 presents the characteristics of patients with ILD. The number of patients who received nivolumab was significantly higher in patients with COP than in those with GGO (*P* = 0.0068). Patients with GGO received anti‐PD‐1 therapy earlier than those with COP (*P* = 0.0055). The expression levels of PD‐L1 on tumor cells were significantly higher in patients with GGO than that in patients with COP (*P* = 0.0496). The median time of onset of ILD was significantly earlier among patients with GGO than among patients with COP (*P* = 0.041). The number of patients who received steroid therapy was significantly higher in the GGO group than in the COP group (*P* = 0.029). Most patients were successfully treated with corticosteroids, but one patient with GGO died from ILD. There were no significant differences in terms of age, sex, smoking status, PS, overall response rate, grade of ILD, other irAEs, or number of patients who received subsequent chemotherapy after anti‐PD‐1 therapy.

**TABLE 2:** Characteristics for patients with ILD

Patient characteristics		COP (n = 16)	GGO (n = 16)	NOS (n = 1)	*P* value^a^
Median age, years (range)		67 (60‐79)	65 (45‐82)	77	.3106^b^
Sex	Female/male	4/12	2/14	1/0	.6539^c^
Smoking status	Current or former	13	16	0	.2258^c^
	Never	3	0	1	
PS	0 or 1	13	11	1	.6851^c^
	≥2	3	4	0	
	Unknown	0	1	0	
Type of PD‐1 therapy	Nivolumab	16	9	0	.0068^c^
	Pembrolizumab	0	7	1	
Line of anti‐PD‐1 therapy, n (%)	1	0	6	1	.0055^d^
	2	12	4	0	
	3	4	6	0	
PD‐L1 expression, n (%)	<1	1	0	0	.0496^d^
	1%‐49%	0	1	0	
	>50%	1	6	0	
	Unknown	14	9	1	
Median treatment cycles (range)		9 (1‐27)	2 (1‐44)	2	.1155^b^
Median time of onset of ILD, days (range)		117 (1‐340)	21 (5‐523)	34	.041^b^
Steroid therapy	Yes	6	13	1	.029^c^
	No	10	3	0	
Response evaluation	ORR	44%	31%	NE	.716^c^
	DCR	81%	50%	NE	.1351^c^
Grade	1‐2	14	9	1	.1134^c^
	≥3	2	7	0	
Other irAEs	Yes	3	6	1	.4331^c^
	No	13	10	0	
Subsequent chemotherapy after PD‐1	Yes	5	7	0	.716^c^
	No	11	9	1	

Abbreviations: COP, cryptogenic organizing pneumonia; DCR, disease control rate; GGO, ground glass opacities; ILD, interstitial lung disease; irAE, immune‐related adverse events; NE, not evaluable; NOS, not otherwise specified; ORR, overall response rate; PD‐1, programmed‐cell death‐1; PD‐L1, PD‐ligand 1; PS, performance status.

^a^ Patient with COP and GGO are compared. Differences between groups were identified using

^b^ student's *t *test

^c^ Fisher's exact test

^d^ Chi‐Square test.

### 3.4 Radiographic patterns of ILD and OS

**FIGURE 3:**
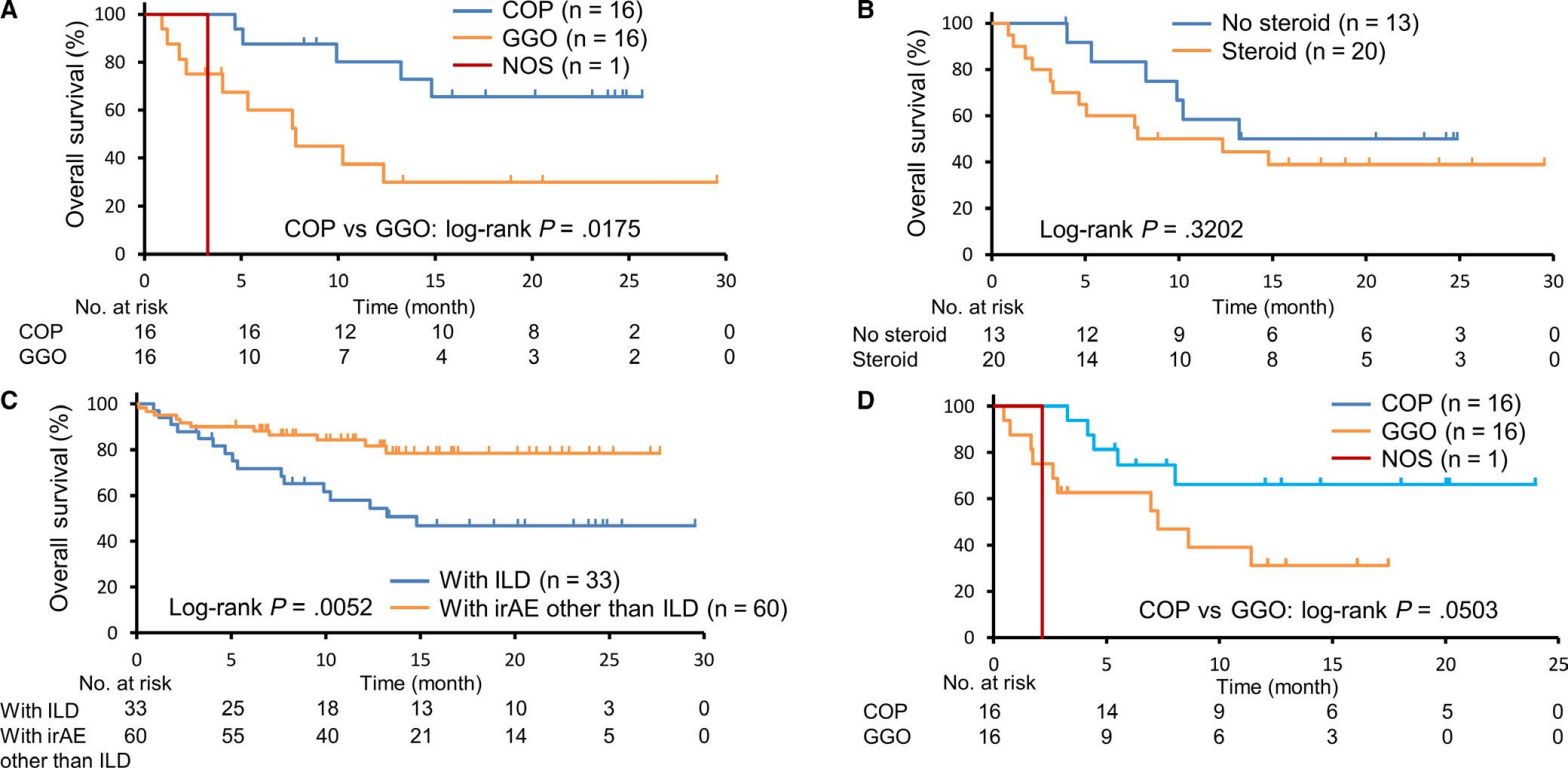
The overall survival curves among patients with COP, GGO, and NOS (A), with or without steroid therapy (B), with ILD or with irAE other than ILD (C). The overall survival curves after the onset of ILD in patients with COP, GGO, and NOS (D). COP, cryptogenic organizing pneumonia; GGO, ground glass opacities; ILD, interstitial lung disease; NOS, not otherwise specified

The Kaplan‐Meier survival curves for patients with COP, GGO and pneumonitis NOS are shown in Figure 3A. The OS was significantly worse among patients with GGO than among those with COP (7.8 months (95% CI: 2.2‐NE) versus not reached (95% CI: 13.2‐NE); *P* = 0.0175). There were no significant difference in PFS between patients with GGO and those with COP (7.6 months (95% CI: 0.9‐NE) versus not reached (95% CI: 2.9‐NE); *P* = 0.1602) (Figure S2). Previous studies indicated that the use of steroids at baseline decreased the clinical benefit of anti‐PD‐1 therapy.^19^ Thus, we evaluated whether steroid administration for the treatment of ILD decreased the antitumor effects of anti‐PD‐1 therapy. Steroid therapies for ILD did not affect the survival times (10.1 months (95% CI: 3.1‐NE) versus not reached (95% CI: 5.3‐NE); *P* = 0.3202) (Figure 3B). Because a correlation between the development of irAEs and a clinical efficacy of anti‐PD‐1 therapy was reported, we assessed OS in patients with irAE other than ILD.^7^, ^8^ The OS for patients with irAE other than ILD was significantly better than that for patients with ILD (not reached (95% CI: NE‐NE) versus 14.8 (95% CI: 7.6‐NE); *P* = 0.0052) (Figure 3C). As shown in Table 2, the median time of onset of ILD was significantly earlier in patients with GGO than in patients with COP; thus, we evaluated OS after the onset of ILD (Figure 3D). The OS after the onset of ILD in patients with GGO tended to be shorter than that in patients with COP (7.3 months (95% CI: 1.7‐NE) versus not reached (95% CI: 5.5‐NE); *P* = 0.0503).

### 3.5 Survival factors

In the multivariate analysis of 231 patients, PS = 1 and ≥2 and GGO were identified as significant predictors of a shorter overall survival time (hazard ratio: 5.4926 (95% CI: 2.3723‐15.9777); *P* < 0.0001, hazard ratio: 21.7533 (95% CI: 8.6118‐66.7892); *P* < 0.0001 and hazard ratio: 7.3029 (95% CI: 2.636‐20.2552); *P* = 0.0003) (Table 3). Similar to a previous report, the presence of any irAE was significantly correlated with prolonged OS (hazard ratio: 0.2692 (95% CI: 0.1117‐0.5455); *P* = 0.0001).

**TABLE 3:** Multivariate analysis by cox proportional hazards model

Characterisitic	Hazard ratio	95% CI	*P* value
Sex (ref = male)			
Female	0.6764	0.3234‐1.3294	.2651
Age (ref = <74)			
>75 years	1.1144	0.6129‐1.9323	.712
Smoking history (ref = never‐smoker)			
Current or former	1.5687	0.683‐3.8502	.2959
Histologic subtype (ref = nonsquamous)			
Squamous	1.1205	0.6548‐1.8915	.674
PS (ref = 0)			
1	5.4926	2.3723‐15.9777	<.0001
>2	21.7533	8.6118‐66.7892	<.0001
Radiologic features (ref = no ILD)			
COP	1.4384	0.4175‐4.5114	.5459
GGO	7.3029	2.636‐20.2552	.0003
irAE (ref = no irAE)			
Yes	0.2692	0.1117‐0.5455	.0001

Abbreviations: CI, confidence interval; COP, cryptogenic organizing pneumonia; GGO, ground glass opacities; ILD, interstitial lung disease; irAE, immune‐related adverse events; PS, performance status.

## 4 DISCUSSION

The current study demonstrated the relationship between the radiologic patterns of ILD caused by anti‐PD‐1 therapy and OS in NSCLC patients. Patients with GGO had a significantly worse survival time than those with COP (Figure 3A). Multivariate analysis also demonstrated that the development of GGO was significantly correlated with poor prognosis (Table 3). In contrast, COP was not correlated with OS (Table 3). Previous studies did not clearly show the association between the development of ILD and patient outcomes after anti‐PD‐1 therapy. Fujimoto et al reported that NSCLC patients with ILD had longer PFS with nivolumab therapy.^10^ On the other hand, Shresh et al showed that the development of ILD decreased survival in NSCLC.^20^ Our study demonstrated that patients with ILD exhibited prolonged PFS; however, patients with ILD had similar OS compared to patients without ILD (Figure 2A and B). Anti‐PD‐1 therapies cause several types of radiologic patterns of ILD, and our results indicate that patients with ILD could be divided into good and poor prognosis groups according to radiologic features. As there is no laboratory test to determine the severity of ILD, we must consider intensive therapy for ILD based on clinical findings, including cough, fever, dyspnea and hypoxemia. Our findings on the correlation between radiologic patterns and clinical outcomes will help physicians make decisions regarding the administration of steroids and immunosuppressants for ILD.

There are several possible explanations for the worse prognosis of patients with GGO. The time of onset of GGO was significantly earlier than that of COP, and the median treatment cycles of anti‐PD‐1 therapy tended to be shorter in patients with GGO than in patients with COP (Table 2). Moreover, 12 out of 16 patients with GGO discontinued anti‐PD‐1 therapy after no more than 3 cycles. The relationship between the discontinuation of PD‐L1/PD‐1 therapy and poor outcome has been indicated previously.^21^, ^22^ Early discontinuation of anti‐PD‐1 therapy could be correlated with worse prognosis in patients with GGO. Another explanation may be the exposure to systemic steroids. Patients with GGO were treated with steroids significantly more frequently than patients with COP (Table 2). Although our data did not demonstrate that steroid therapy decrease OS among patients with ILD, previous studies have demonstrated that patients treated with systemic steroid during the first cycle of nivolumab had a shorter survival time (Figure 3B).^19^

Previous studies have also demonstrated that patients with irAEs have a more favorable prognosis than patients without irAEs.^7^, ^8^ Similar to these studies, the current study showed that patients with irAEs other than ILD had significantly better survival times compared to patients with ILD (Figure 3C).

The limitations of the current study include a relatively small number of patients with ILD and its retrospective nature. Although all medical records of consecutive NSCLC patients receiving anti‐PD‐1 therapy were assessed, radiology review was performed with only clinically determined ILD cases, not all cases treated with anti‐PD‐1 therapy. In our study, no patients were diagnosed with ILD based on pathological evidence.

To the best of our knowledge, this is the first report to demonstrate the correlation between radiologic features of ILD and OS in NSCLC patients. Our findings suggest that patients with GGO require intensive therapy more often than patients with COP. A prospective study is required to establish the treatment strategy for ILD according to radiologic patterns. The fact that some patients with GGO responded to anti‐PD‐1 therapy indicates that damage to normal lung tissues might be different even in patients with similar radiologic features of ILD. Evaluation of lung tissues from patients with ILD and assessment of the correlation between radiologic patterns and histology are warranted.

## CONFLICT OF INTEREST

Dr Watanabe received lecture fees from Eli Lilly, Pfizer, Novartis Pharma, AstraZeneca, Chugai Pharma, Bristol‐Myers, Boehringer Ingelheim, MSD, Ono Pharmaceutical, Daiichi Sankyo and Taiho Pharmaceutical; Dr Ota received lecture fees from Boehringer Ingelheim, MSD, Eli Lilly, AstraZeneca and Chugai Pharma; Dr Hayashi received lecture fees from AstraZeneca, Ono Pharmaceutical, Boehringer Ingelheim, Daiichi Sankyo, Taiho Pharmaceutical and Actelion Pharmaceuticals Japan; Dr Ishikawa received lecture fees from Daiichi Sankyo, Bayer, Nihon Medi‐Physics and AstraZeneca; Dr Shoji received lecture fees from AstraZeneca, Chugai Pharma, Taiho Pharmaceutical and MSD; Dr Nozaki received lecture fees from Bristol‐Myers, Pfizer and Novartis; Dr Ichikawa received lecture fees from AstraZeneca, Chugai Pharma, Bristol‐Myers, Boehringer Ingelheim, Ono Pharmaceutical and Taiho Pharmaceutical; Dr Miyabayashi received lecture fees from Chugai Pharma, Ono Pharmaceutical, AstraZeneca and Actelion Pharmaceuticals; Dr Miura received lecture fees from Bristol‐Myers, Ono Pharmaceutical, Boehringer Ingelheim, Eli Lilly, MSD, Chugai Pharma, AstraZeneca, Taiho Pharmaceutical, Kyowa Hakko Kirin and Mochida Pharmaceutical; Dr Tanaka received lecture fees and grants from Bristol‐Myers, Eli Lilly, MSD, Taiho Pharmaceutical, Pfizer, Chugai Pharma, Ono Pharmaceutical, AstraZeneca, Boehringer Ingelheim, lecture fees from Novartis and grant from Merck Serono; Dr Abe received lecture fees from AstraZeneca, Chugai Pharma, Boehringer Ingelheim, Eli Lilly, Taiho Pharmaceutical and Bristol‐Myers; Dr Okajima received lecture fees from AstraZeneca, Ono Pharmaceutical, Bristol‐Myers, Boehringer Ingelheim, MSD and Taiho Pharmaceutical; Dr Terada received lecture fees from AstraZeneca, Chugai Pharma, Bristol‐Myers, Boehringer Ingelheim, Taiho Pharmaceutical, MSD and Ono Pharmaceutical; Dr Ishida received lecture fees from Boehringer Ingelheim, Bristol‐Myers, Chugai Pharma, Eli Lilly and AstraZeneca; Dr Iwashima received lecture fees from AstraZeneca, Chugai Pharma, Bristol‐Myers, Boehringer Ingelheim, Taiho Pharmaceutical, MSD, Ono Pharmaceutical, Novartis, Actelion Pharmaceuticals, Torii Pharmaceutical and KYORIN Pharmaceutical; Dr Sato received lecture fees from AstraZeneca, Boehringer Ingelheim, Ono Pharmaceutical, MSD and Pfizer; Dr Yoshizawa received lecture fees from AstraZeneca, Boehringer Ingelheim, Taiho Pharmaceutical, Chugai Pharma and Ono Pharmaceutical; Dr Kikuchi received grant and lecture fees from Chugai Pharma, Boehringer Ingelheim, Eli Lilly, MSD, Taiho Pharmaceutical, Daiichi Sankyo, Ono Pharmaceutical AstraZeneca, Shionogi, TEIJIN PHARMA and KYORIN Pharmaceutical, and lecture fees from Astellas Pharma, Bristol‐Myers, Pfizer, Taisho Toyama Pharmaceutical, Janssen Pharmaceutical, Japan BCG Laboratory Novartis, Mylan NV and Roche Diagnostics. All remaining authors have declared no conflicts of interest.

## AUTHORS' CONTRIBUTIONS

All authors contributed to conceptualization, writing‐review and editing, and final approval of the article. Additional contributions are as follows: **Satoshi Watanabe**: Methodology, formal analysis, and writing–original draft. **Masachika Hayashi and Hiroyuki Ishikawa:** Data analysis and interpretation. **Takeshi Ota, Aya Otsubo, Satoshi Shoji, Koichiro Nozaki, Kosuke Ichikawa, Rie Kondo, Takao Miyabayashi, Satoru Miura, Hiroshi Tanaka, Tetsuya Abe, Masaaki Okajima, Masaki Terada, Takashi Ishida, Akira Iwashima, Kazuhiro Sato and Hirohisa Yoshizawa:** Collection of data. **Toshiaki Kikuchi:** Supervision.

## Supporting information

Fig S1

Fig S2

## Data Availability

The data in this study are available if the request is acceptable by the corresponding author.
