# Supplementary figures and images for: Prognostic significance of the radiologic features of pneumonitis induced by anti‐PD‐1 therapy
Source: Cancer Med. 2020 Mar 9;9(9):3070–7. doi: 10.1002/cam4.2974 (PMC7196069; doi:10.1002/cam4.2974)

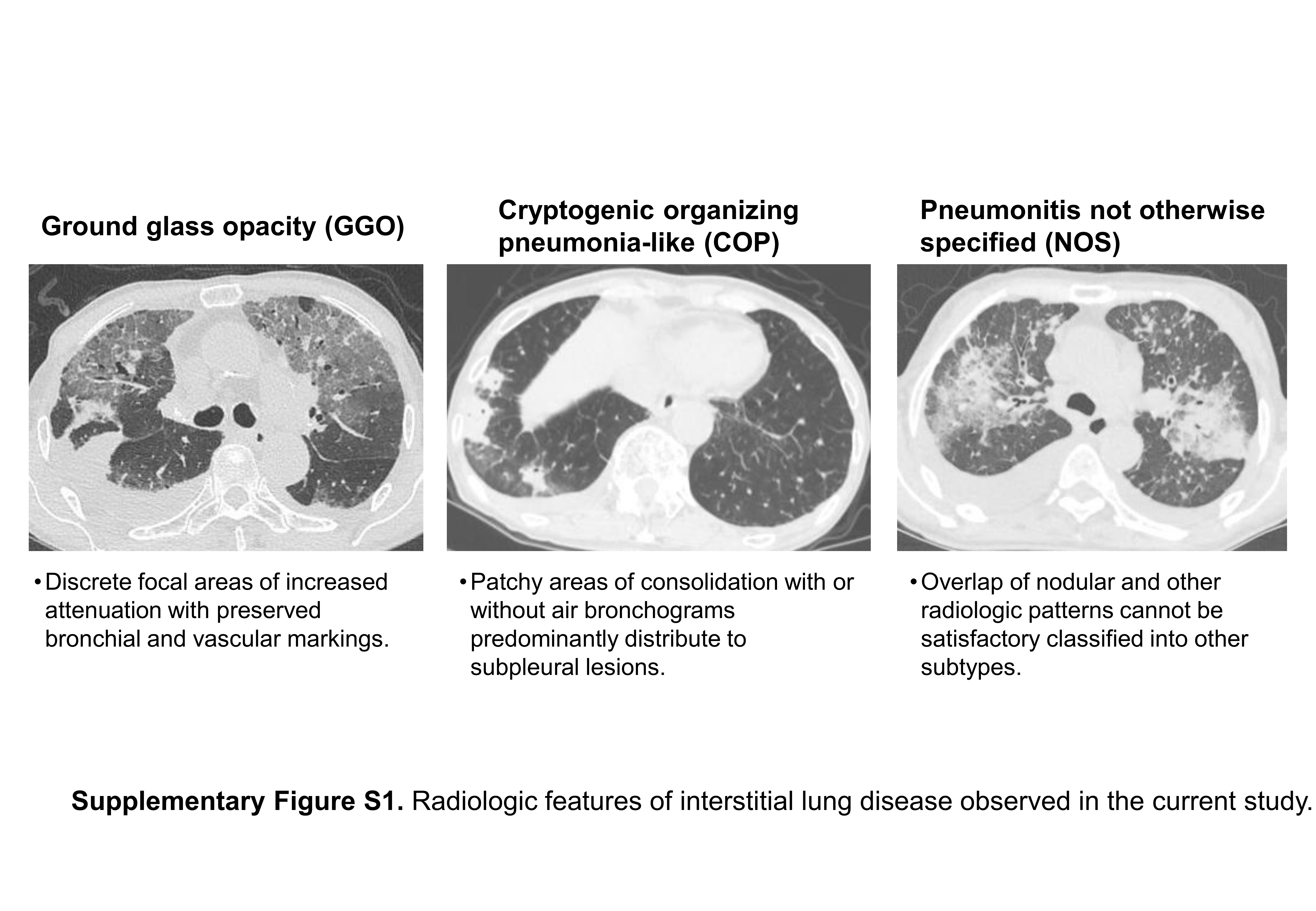

Supplement: Supplementary file 1 — Fig S1 [file CAM4-9-3070-s001.tif]

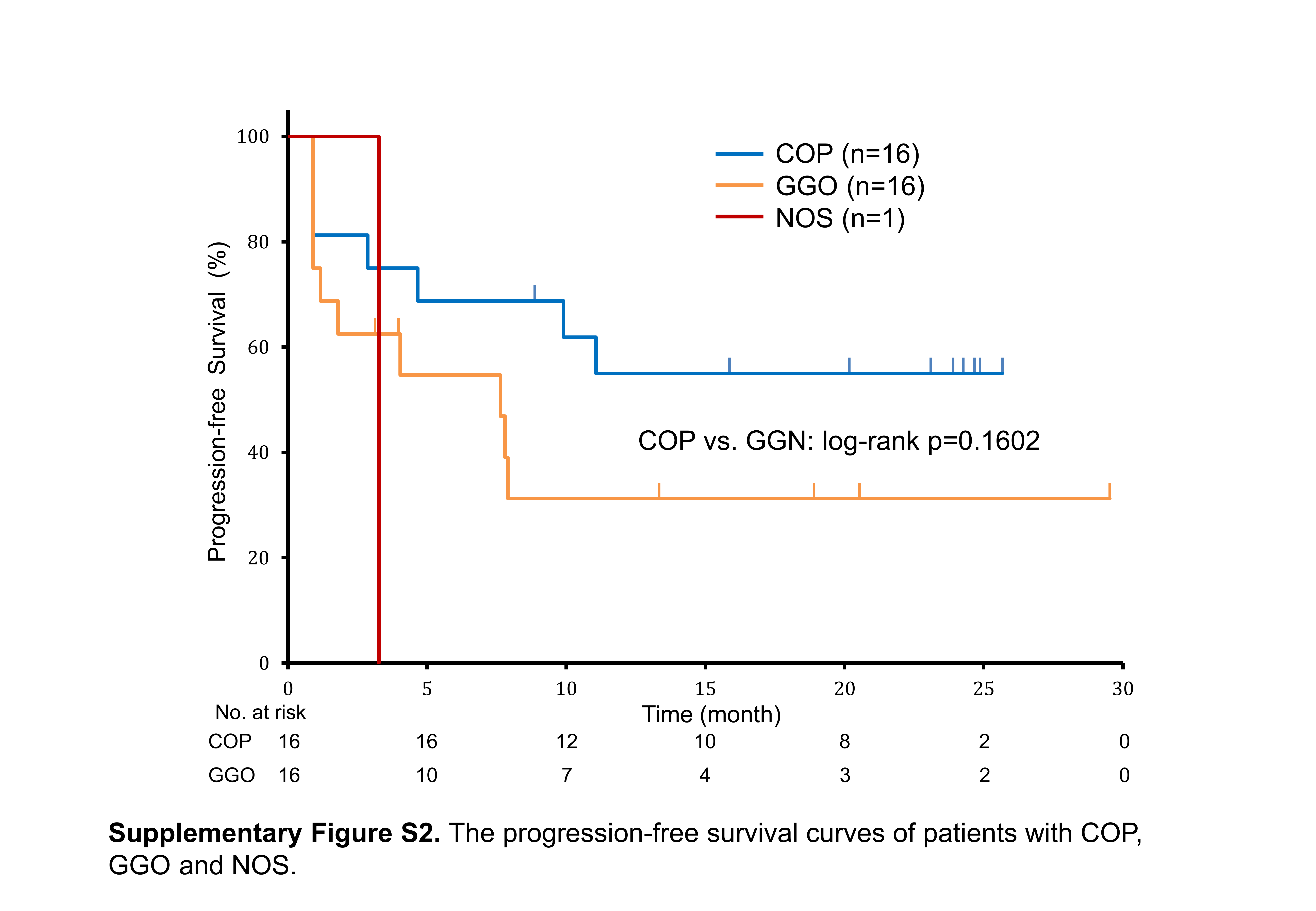

Supplement: Supplementary file 2 — Fig S2 [file CAM4-9-3070-s002.tif]
